# *In vitro* antibacterial activity of medicinal plants against biofilm-forming methicillin-resistant *Staphylococcus aureus*: efficacy of *Moringa stenopetala* and *Rosmarinus officinalis* extracts

**DOI:** 10.1016/j.heliyon.2020.e03303

**Published:** 2020-01-27

**Authors:** Aseer Manilal, Kuzhunellil Raghavanpillai Sabu, Misgun Shewangizaw, Addis Aklilu, Mohammed Seid, Behailu Merdikios, Behailu Tsegaye

**Affiliations:** a Department of Medical Laboratory Science, College of Medicine and Health Sciences, Arba Minch University, Arba Minch, Ethiopia; b Department of Chemistry, College of Life Sciences, Arba Minch University, Arba Minch, Ethiopia; c Department of Public Health, College of Medicine and Health Sciences, Arba Minch University, Arba Minch, Ethiopia; d Department of Biomedical Science, College of Medicine and Health Sciences, Arba Minch University, Arba Minch, Ethiopia

**Keywords:** Microbiology, Biotechnology, Metabolite, Medical microbiology, Antimicrobial, MRSA, Antibacterial activity, Anti-biofilm activity, *Moringa stenopetala*, Plant extract

## Abstract

The prevalence of methicillin-resistant *Staphylococcus aureus* (MRSA) is slowly rising in Ethiopia for the past few decades. Therefore, novel classes of antibiotics are indispensable to combat the increased incidence of newly emerging multidrug-resistant bacteria like MRSA. Terrestrial flora is considered as a reservoir of novel bioactive secondary metabolites as they have provided us with the largest array of natural products. In this background, the present study is intended to evaluate the *in-vitro* antibacterial efficacy of five medicinal plants (*Ocimum lamiifolium* Hochst. ex Benth.*, Rosmarinus officinalis* L*, Catharanthus roseus* Linn.*, Azadirachta indica* A. Juss *and Moringa stenopetala* Bac) against a panel of seven biofilm-forming MRSA. The leaves of the plants were extracted in organic solvents of varying polarity and the resultant crude extracts of respective medicinal plants were inspected for their antimicrobial activity by well diffusion technique. Minimum inhibitory concentrations (MIC) and minimum bactericidal concentrations (MBC) of the plant extracts against MRSA were determined by the broth dilution method. Besides, an anti-biofilm assay of the most potent plant extract was also performed, after which its chemical constituents were delineated by combined Gas Chromatographic and Mass Spectroscopic profiling (GC-MS). The results revealed that, of the five plants, three species including *M. stenopetala, R. officinalis,* and *O. lamifolium* exhibited significant antibacterial activity. Organic solvents with high and medium polarity were excellent in extracting antimicrobials compared to nonpolar solvents. The broadest and highest rank of activity was observed in the crude ethanolic extract of *M. stenopetala.* Based on the MIC/MBC ratio, the crude ethanolic extract of *M. stenopetala* was determined to be bacteriostatic. Anti-biofilm assay showed that the extract of *M. stenopetala* fairly inhibited the growth of MRSA in the preformed biofilm matrix. The GC-MS analysis of *M. stenopetala* revealed the presence of twelve compounds with antimicrobial activity. The present study provides new insight into the development of novel drug leads to the management of MRSA.

## 1 Introduction

Among the diverse multidrug-resistant bacteria, methicillin-resistant *Staphylococcus aureus* is probably the best-known resistant bacterium that has riveted intense scientific and political interest globally due to the limited spectrum of antibiotics for its effective treatment (Easton et al., 2009; WHO, 2017). Recent estimates of the World Health Organization revealed that the combined prevalence of MRSA in a diverse population of the African region is between 12 and 80 % (WHO, 2014). In Ethiopia, the prevalence of MRSA is slowly rising in the past few decades. In most of the hospitals, antibiotic treatment is not streamlined as per the microbiological culture data. Consequently, MRSA strains are becoming resistant not only to β-lactams but also to multiple antimicrobial agents, such as macrolides, aminoglycosides, and fluoroquinolones (Eshetie et al., 2016; Mama et al., 2018). From this standpoint, it is inevitable to develop novel antibiotics with negligible side effects and hence no incidence of specific drug resistance.

Since time immemorial, the plant kingdom has catered to the need of humans by providing a variety of drugs. Among them, terrestrial flora constitutes a prolific source of bioactive constituents. Plants synthesize a wide variety of metabolites to safeguard them and to maintain homeostasis in their environment. Investigations on naturally occurring antibiotic compounds from terrestrial plants have been studied for more than a century (Berdy, 2005). Hitherto, more than 200,000 natural products have been recorded from diverse species of flora (Lei Yang et al., 2016). It has been purported that secondary metabolites from medicinal plants have minimal adverse reactions and also can repress the growth of pathogens by diverse mechanisms than the currently used synthetic antibiotics. Often these secondary metabolites differ among the species of plants with respect to their quantity, diversity and biological activities (Manilal et al., 2011). The abundance and diversity of high-value secondary metabolites in plants render them a remarkable source of antibacterial agents for pharmaceutical industries. About 25–28 % of modern medicines are formulated from the diverse secondary metabolites of higher plants (Samuelsson, 2004). It is envisaged that the plant-derived drug market will grow from 29.3 billion US dollars in 2017 to around 39.2 billion by 2022 (Stefanovic, 2018). Therefore, the screening of medicinal plants could facilitate the discovery of novel and effective natural products suitable for biopharmaceutical sectors.

Medicinal plants still play a paramount role in the health care system of indigenous communities in Ethiopia, particularly those in remote rural settings, which are beyond the reach of government health service. In many rural regions of Ethiopia, medicinal plants are the most easily accessible resource to the community, making it the most preferred option. However, researches on many of these medicinal plants are still in the stage of infancy. Approximately 6,500 species of plants are documented from different regions of Ethiopia, and it ranks six among the countries in Africa in the number of recorded endemic plant species (Convention on Biological Diversity, 2009). These vast diversities of terrestrial plants are the reservoir of untapped secondary metabolites, which might be a potential source of drugs for the future (Ameya et al., 2019). Medicinal plants such as *O. lamiifolium, R. officinalis, C. roseus, A. indica*, and *M. stenopetala* are widely distributed in Ethiopia. All these plants are used for the preparation of traditional medicines and few of them for culinary purposes. A perusal of the literature indicated that the antimicrobial activities of these medicinal plants against bacteria have been extensively investigated by a number of researchers (Geyid et al., 2005; Naz et al., 2015; Lulekal et al., 2014; Mekonnen et al., 2016; Meressa, 2017; Sahalie et al., 2018). Nevertheless, studies on the effect of Ethiopian medicinal plants against biofilm-forming MRSA sourced from human immunodeficiency virus (HIV) infected patients are not done so far. Hence, the present study was intended to screen five medicinal plants, against biofilm-forming MRSA isolated from HIV patients.

## 2 Materials and methods

### 2.1 Collection and extraction of secondary metabolites from plants

Field collection of tender foliage of five medicinal plants viz., *Ocimum lamiifolium, Rosmarinus officinalis, Catharanthus roseus, Azadirachta indica*, and *Moringa stenopetala* were done from the Arba Minch expanse (Table 1). The plant specimens were identified to the genus and species level with the aid of eminent plant taxonomist (Dr. Remesh Moochikkal, Jazan University, Kingdom of Saudi Arabia). The secondary metabolites were isolated employing an immersion extraction process using appropriate solvent systems as described in detail previously (Manilal and Idhayadhulla, 2014). Fresh leaves were separately extracted in four different solvents (High-Performance Liquid Chromatography grade diethyl ether, ethyl acetate, methanol, and ethanol procured from Fisher Scientific co.) using mortar and pestle. Afterward, the ground leaves were kept immersed in respective solvents for two days to permit full extraction. The resultant extracts were filtered and concentrated to near dryness in a hot air oven at 40 °C, weighed and placed in a refrigerator at 4 °C until the antimicrobial assay.

**Table 1:** List of plants used for the anti-staphylococcal activity.

Voucher specimen No.	Plant species	Family	Vernacular name	Parts used
*AMP10*	*Ocimum lamiifolium*	Lamiaceae	Damakese	Leaves
AMP11	*Rosmarinus officinalis*	Lamiaceae	Sigametbesha	Leaves
AMP12	*Catharanthus roseus*	Apocynaceae	Abeba	Leaves
AMP13	*Azadirachta indica*	Meliaceae	Neem	Leaves
AMP14	*Moringa stenopetala*	Moringinaceae	Shifera	Leaves

### 2.2 *In vitro* anti-staphylococcal assay

#### 2.2.1 Microbial test cultures

Seven strains of MRSA, isolated from HIV infected patients were used (Manilal et al., 2019). These MRSA isolates were established as strong biofilm producers and deposited in Medical Microbiology and Parasitology Laboratory, College of Medicine and Health Sciences, Arba Minch University, Arba Minch (Manilal et al., 2019). The resistance pattern towards cefoxitin and biofilm-forming potentials of these isolates were re-confirmed as per the methodology described elsewhere (Manilal et al., 2019). MRSA strains were maintained on mannitol salt agar (MSA) at 37 °C. The purity of MRSA isolates was subsequently checked by the inspection of colony morphology, cell morphology (Gram-stained), and by biochemical tests (catalase and coagulase) following standard procedures. A reference standard of *S. aureus* (ATCC 25923) was also used as a control to validate the biochemical results.

#### 2.2.2 Agar diffusion assay

To examine the anti-staphylococcal activity, the plant extracts were evaluated against a battery of MRSA isolated from HIV infected patients. The crude extracts of respective plant spp. were inspected for antibacterial activity utilizing the agar diffusion assay as described in our previous study (Manilal and Idhayadhulla, 2014). In short, sterile Mueller Hinton agar was dispensed into Petri plates and uniformly swabbed with 100 μl of a suspension containing 10^8^ colony-forming unit/mL of appropriate strains of MRSA. For the antimicrobial assay, inoculums were prepared from overnight cultures by the direct colony method. Discrete colonies were picked up directly from the plate with a sterile wire loop and suspended into sterile 0.85 % saline. The turbidity of suspension to be inoculated was adjusted in line with 0.5 Mc Farland standard. Afterward, test organisms were uniformly swabbed over the Mueller-Hinton agar (Hi-Media, Mumbai) surface. Five-millimeter diameter well was made on the seeded surface using a sterile cork borer and 100 μL of the appropriate extract of a known concentration corresponding to 2 mg/ml (prepared by dissolving 100 mg of the dried crude extract in 10 ml of respective solvents) was placed inside the wells. Two commercially available antibiotics discs (clindamycin [2 μg]; vancomycin (30 μg)] (Himedia®, Mumbai)) served as positive controls. Organic solvents used for the extraction were used as negative controls. The assays were conducted in triplicate to validate the findings statistically. The Petri plates were incubated for 24 h at 37 °C and the inhibitory activity was measured by calculating the diameter of a clear zone around the well using ruler scale. The overall activity indices were calculated using the following formula:

$$\text{Overall}\;\text{Activity}\;\text{Index}\;\text{(OAI)}=\frac{\text{No.}\;\text{of}\;\text{solvent}\;\text{extracts}\;\text{with}\;\text{a}\;\text{zone}\;\text{of}\;\text{inhibition}\;\geq 8.0\;\text{mm}}{\text{Total}\;\text{No.}\;\text{of}\;\text{strains}\;\text{of}\;\text{MRSA}\;\text{tested}}\text{x}100$$

#### 2.2.3 Determination of mechanisms of antibiosis

The broth dilution method was employed to determine the minimum inhibitory concentration of highly active crude plant extracts (Ameya et al., 2019). The dried ethanolic extracts of plants, aliquoted in phosphate-buffered saline (pH 7.2) were used as the test solutions. The dosing range of plant extracts was computed by a factor of 2 (antilog 0.3) to obtain a final dose range between 125 and 8000 μg per milliliter in Mueller Hinton broth supplemented with 4 % sodium chloride. Afterward, each tube was inoculated with 100 μL of a fresh overnight culture of appropriate MRSA isolates and incubated at 37 °C for 24 h. Based on the results of preliminary experiments, the concentration range of plant extracts was further narrowed down to obtain the specific MIC values (125 and 8000 μg per milliliter). MICs were recorded as the lowest concentration of respective plant extracts that prevented visible growth as indicated by the absence of turbidity in line with the control. To measure the minimum bactericidal concentration, MIC cultures were seeded (10 μl) on Mueller–Hinton agar supplemented with 4 % sodium chloride and incubated for 24 h at 37 °C. MBC was defined as the concentration which exhibits zero growth of the colonies compared to the culture of the initial inoculum of the same strain.

### 2.3 Anti-biofilm assay

The highly active plant, *M. stenopetala* was subjected to anti-biofilm assay. The preliminary experiment was performed by qualitatively analyzing the viability of MRSA embedded in the preformed biofilm matrix in culture tubes with *M. stenopetala* extracts of particular concentrations, with reference to controls. Three strains of strong biofilm forming MRSA were cultured overnight in trypticase soy broth (TSB) supplemented with 1 % glucose and the biofilm formation rate was qualitatively re-confirmed as per Manilal et al. (2019). For the assay, fresh overnight poly-culture (100 μl/strain) of three strong biofilm-forming MRSA (1×10^8^ colony forming units) were inoculated into 2 ml culture tubes containing TSB and incubated aerobically for 24 h at 37 °C under the static condition to permit the biofilm formation (Manilal et al., 2019). Afterward, different dose levels (ranging between 124.8 and 1000 μg per milliliter) of dried ethanolic extract prepared in phosphate-buffered saline (pH 7.2) were added to each culture tube. Parallelly, negative control (culture tube with phosphate-buffered saline) and positive control (culture tube with 10 % NaOCl) was used to validate the inferences. After 24 h of the incubation period, planktonic cells and consumed media were decanted and rinsed thrice with sterile physiological saline. Preliminary identification of anti-biofilm activity was done macroscopically based on the absence of slime formation at the air-liquid interface and also at the bottom of culture tubes compared to that of controls. To evaluate the anti-biofilm activity, the biofilm matrix adhered to the inner wall and the bottom of each culture tubes were collected using a sterile swab wetted with normal saline (0.85 % NaCl). Afterward, the respective swabs were directly inoculated into MSA and incubated for 24 h at 37 °C. A positive result was recorded corresponding to the respective concentrations of plant extract that totally inhibited the visible growth of MRSA embedded in the preformed biofilm matrix. The presence of MRSA growth was considered as negative. Tests were conducted in triplicates and the result was expressed as activity in terms of μg of extract per milliliter.

### 2.4 Gas Chromatographic and Mass Spectroscopic analysis

Shimadzu QP-2010 GC-MS system equipped with a capillary column (inner diameter 0.25 mm and length 30 m) was used to analyze the constituents of the most active extract, that of *M. stenopetala* (Ameya et al., 2019)*.* The temperature of GC oven was kept at 100 °C for two minutes and was further programmed to 280 °C at the rate of 10 °C/min and then kept at 280 °C for 13 min. The split ratio was 1: 25 and the injection volume was 2 μl. The injection port and detector port temperatures were 200 °C and 240 °C respectively. The GC-MS electron ionization mode was 150 eV. The mass scan ranged from m/z 40–400 amu. The peaks of the gas chromatogram were subjected to mass spectral analysis. The active constituents were identified based on the retention indices and by the comparison of mass spectra with the National Institute of Standards and Technology (NIST) library of mass spectral data.

### 2.5 Data analysis

The data were described as mean ± standard deviation (S.D.) using SPSS for Windows version 20. (Statistical Package for Social Services, Chicago, IL, USA).

## 3 Results

### 3.1 Anti-staphylococcal activity

**Figure 1:**
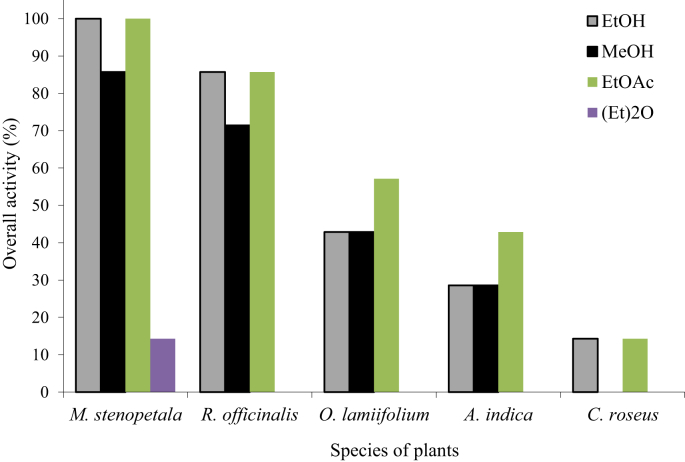
Overall inhibitory activity index of different solvent extracts of five plants. [EtOH: Ethanol; EtOAc: Ethyl acetate; MeOH: Methanol; (Et)_2_O: Diethyl ether].

The results of primary screening showed that all the plants including *O. lamiifolium, R. officinalis, C. roseus, A. indica*, and *M. stenopetala* showed antibacterial activities of varying degrees. All five species of plants showed antibiosis against at least one strain of MRSA tested. The results revealed that three species of plants including *M. stenopetala, R. officinalis,* and *O. lamifolium* exhibited significant antibacterial activity. The overall activity index of all crude extracts of five plants against the MRSA is shown in Figure 1. It was observed that the overall activity index of *M. stenopetala* ranged between 85.7 and 100 %, whereas the extract of *R. officinalis* showed activity indices ranging between 71.4 and 85.7 % followed by *O. lamifolium* (42.8–57.1 %).

Relative analysis of the anti-staphylococcal activity of different crude extracts obtained from five plant species showed that the activity was substantially higher for ethanolic extracts followed by ethyl acetate and methanolic extracts. Particularly, the crude ethanolic extract of *M. stenopetala* showed the highest zones of inhibition against all the MRSA tested and was even comparable with the activities of clindamycin and vancomycin (positive controls). However, extracts obtained using methanol and ethyl acetate showed varying ranks of activity towards the MRSA tested. Under this experimental condition, no obvious zone of inhibition was observed for diethyl ether extracts and negative controls. For this reason, those solvent extracts were excluded from further studies. As expected, all the MRSA showed higher sensitivity towards vancomycin and clindamycin. The inhibition zone of *M. stenopetala* extract was more or less similar to the zone of inhibition produced by reference antibiotics.

**Figure 2:**
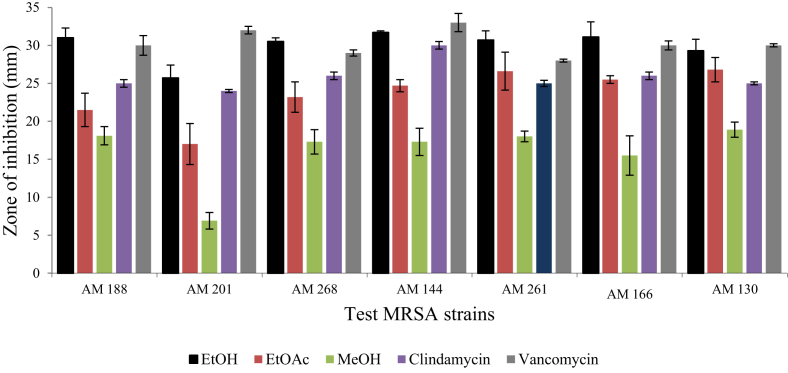
Anti-staphylococcal activity of different solvent extracts of *M. stenopetala* and comparable with the activities of clindamycin and vancomycin (positive controls).

**Figure 3:**
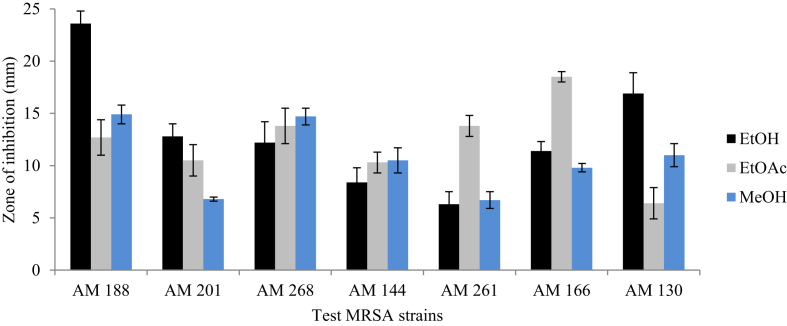
Anti-staphylococcal activity of different solvent extracts of *R. officinalis*.

**Figure 4:**
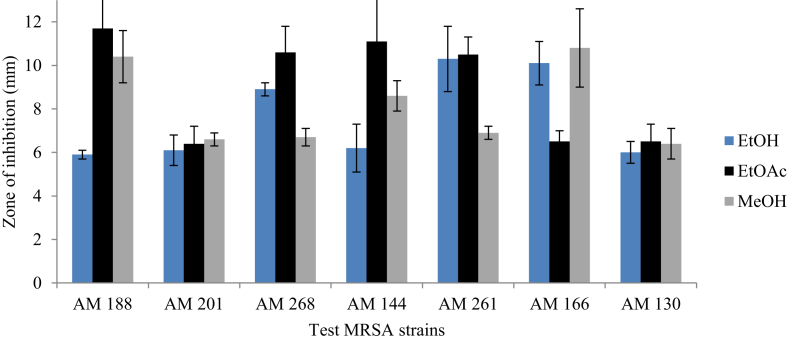
Anti-staphylococcal activity of different solvent extracts of *O. lamiifolium*.

**Figure 5:**
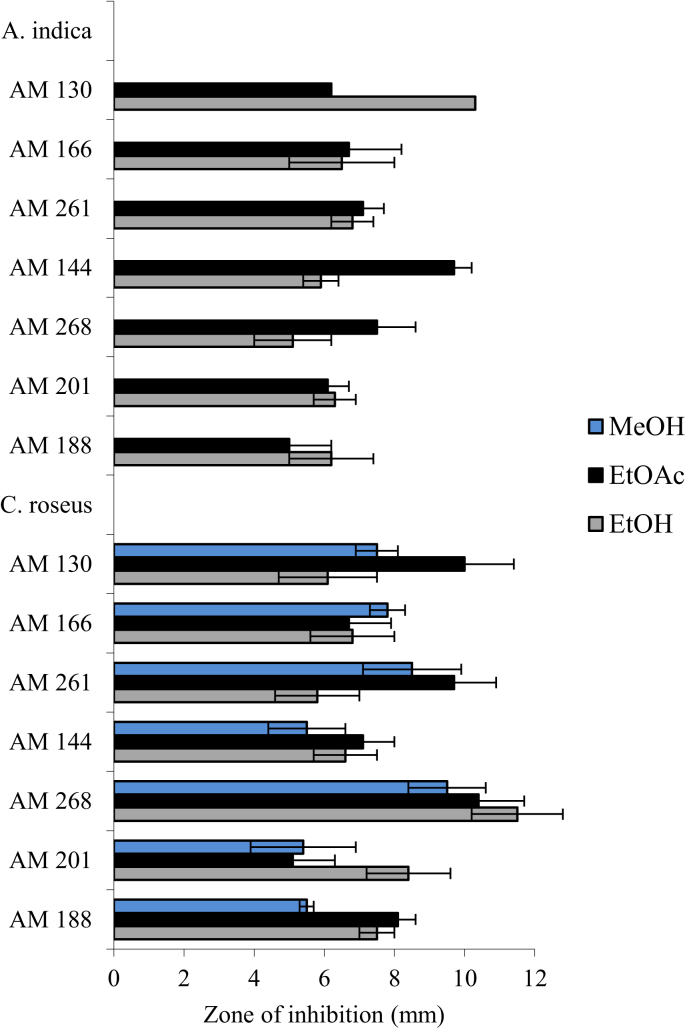
Anti-staphylococcal activities of different solvent extracts of *A. indica* and *C. roseus*.

Among the five plants screened*, M. stenopetala* showed the highest and the widest spectrum of activity (Figure 2). It was found that crude ethanolic and methanolic extracts of *M. stenopetala* efficiently inhibited the growth of all MRSA isolates to different ranges. Consequently, the ethanolic extract of *M. stenopetala* produced an inhibitory zone in the range of 25.7 ± 1.7 mm and 31.7 ± 0.2 mm. Likewise, the ethyl acetate extract of *M. stenopetala* showed an inhibitory zone ranging between 17 ± 2.7 mm and 26.8 ± 1.6 mm. The second most effective plant was *R. officinalis* (Figure 3). Its ethanolic extract exhibited an inhibitory zone ranging between 8.4 ± 1.4 mm and 23.6 ± 1.2 mm, while in the case of *O. lamiifolium*, ethyl acetate was the best solvent that displayed an inhibitory zone ranging between 10.5 ± 0.8 mm and 8.9 ± 0.3 mm (Figure 4). However, other plants such as *C. roseus* and *A. indica* presented only lower degrees of activity (Figure 5). Based on the wide spectrum of activity, *M. stenopetala* and *R. officinalis* were further chosen for determining the minimum inhibitory concentration.

### 3.2 Mechanism of antibiosis

The MIC and MBC values are presented in Table 2. The MIC values of *M. stenopetala* against the MRSA ranged between 125 and 250 μg/ml and its corresponding MBC values ranged between 500 and 1000 μg/ml. The lowest MIC value recorded against three MRSA isolates was 125 μg/ml and the corresponding MBC value was 500 μg/ml. In the case of *R. officinalis,* the MIC values were recorded in the range of 1000–2000 μg/ml and the respective MBC values registered were in the range of 4000–8000 μg/ml. The overall results indicate that anti-bacterial constituents can be detected in *M. stenopetala* and *R. officinalis*.

**Table 2:** Minimum inhibitory concentration and minimum bactericidal concentration of ethanolic extract of *M. stenopetala* and *R. officinalis* against MRSA isolates.

MRSA Accession number	*M. stenopetala*			*R. officinalis*		
	MIC (μg/ml)	MBC (μg/ml)	MBC/MIC	MIC (μg/ml)	MBC (μg/ml)	MBC/MIC
AM 201	125	500	4	1000	4000	4
AM 261	250	1000	4	2000	8000	4
AM 130	125	500	4	1000	4000	4
AM 166	250	1000	4	2000	8000	4
AM 188	125	500	4	1000	4000	4

### 3.3 Anti-biofilm activity of *M. stenopetala* extract

The concentration of plant extract that completely prevented the visible growth/colonies of MRSA embedded in the biofilm matrix can be considered as an indication of biofilm inhibition. It has been revealed that the ethanolic extract of *M. stenopetala* showed considerable anti-biofilm forming activity against the MRSA in comparison with the negative control. The biofilm production of poly-culture of MRSA was significantly inhibited by 1000 μg/ml of the plant extract.

### 3.4 GC-MS analysis of *M. stenopetala* extract

The highest activity exhibited by ethanolic extracts of *M. stenopetala* prompted us to do the further chemical investigation by GC-MS analysis. A high-resolution mass spectrometer equipped with a data system in combination with GC was used for the chemical analysis of an ethanolic extract of the plant. The spectra of the unknown constituents were compared with known constituents deposited in the NIST library. Chemical constituents of crude extract analyzed by GC-MS were found to contain a mixture of fatty acids with volatile compounds. The identified compounds constitute 99.25 % of the total extracts. Twenty-four peaks were observed in the case of *M. stenopetala*. The retention time, molecular weight and the bioactivity of the compounds corresponding to the 24 peaks are presented in Table 3. Major compounds identified were different types of esters (23.8 %), olefinic alcohols (15.5 %), alcohols (15.4 %), fatty acids (9.4 %), aldehydes (9.4 %), diterpenes (7.9 %), substituted phenol (7 %) and substituted polysaccharides (3.39 %). Minor, but biologically active compounds include dioxane (3.4 %), ketones (2.31 %) and siloxane (1.5 %). Among the 24 peaks, a few compounds including oleic acid, tetradecanoic acid, octadecanoic acid, n-hexadecanoic acid, phenol, 2,4-bis(1,1-dimethylethyl), beta.-l- rhamnopyranoside, 5-o-acetyl-thio-octyl, di-(2-ethylhexyl)phthalate, phytol were the prominent antimicrobial compounds.

**Table 3:** The phytoconstituents identified from the ethanolic extract of *M. stenopetala* by GC-MS analysis.

RT	Compounds	Molecular weight	Peak area (%)	Functional group
3.21	2,4-Dihydroxy-2,5-dimethyl-3(2H)-furan-3-one	144	0.01	Hydroxy ketone
3.93	p-Dioxane, 2,5-dimethyl-3-methylene	128	3.4	Alkylated dioxane
5.9	Acetate, 2-hydroxy-2-(3-chloro-4,5-dihydro-5-isoxazolyl)-, ethy	207	2.6	Ester (hydroxyl-choloro)
7.5	4H-Pyran-4-one, 2,3-dihydro-3,5-dihydroxy-6-methyl	144	2.3	Di-hydroxy ketone
9.1	2,6-Dimethylbenzaldehyde	134	9.4	Aldehyde
10.8	1-Undecanol	172	0.5	Alcohol
12.3	Phenol, 2,4-bis(1,1-dimethylethyl)	206	7.08	Alkyl phenol
13.3	Cyclopropanetetradecanoic acid, 2-octyl-, methyl ester	394	4.8	Methyl ester
15.7	Propanoic acid, 3,3′-thiobis-, diethyl ester	234	4.1	Ethyl ester (Sulfur containing)
17.4	1-Hexadecanol	242	14.9	Alcohol
18.7	2-Propenoic acid, tridecyl ester	254	3.5	Ester (Tridecyl)
19.7	l-Gala-l-ido-octose	240	0.39	Substituted polysaccharide
21.2	1-(3-Benzyl-2-thioureido)-1-deoxy-.beta.-d-glucopyranose 2,3,4,	496	1.5	Substituted polysaccharide
22.3	Tetradecanoic acid	228	0.96	Free fatty acid
25.3	Cyclo propane dodecanoic acid, 2-octyl-, methyl ester	366	1.039	Methyl ester
27.1	Phytol	296	7.94	Diterpene
27.5	2-(2′,4′,4′,6′,6′,8′,8′-Heptamethyltetrasiloxane-2′-yloxy)-2,4,4∗	652	1.538	Siloxane
28.4	Oleic Acid	282	5.7	Free fatty acid
28.9	n-Hexadecanoic acid	256	1.9	Free fatty acid
30.6	Propanoic acid, 3-mercapto-, dodecyl ester	274	5.1	Ester (substituted with long chain alkyl group)
33.7	2-Methyl-Z,Z-3,13-octadecadienol	280	15.5	Olefinic alcohol
34.8	Octadecanoic acid	284	0.90	Free fatty acids
38.04	Di-(2-Ethylhexyl)phthalate	390	2.70	Ester
39.6	beta.-l- *rhamnopyranoside*, 5-O-acetyl-thio-octyl	334	1.5	Alkyl substituted polysaccharide

RT- Retention Time (minutes).

∗ It is suspected that the source of siloxane is the capillary column used and not the plant extract.

## 4 Discussion

### 4.1 Primary screening

The results of our primary screening by agar well diffusion, as mentioned earlier revealed that among the five species of plants studied, two of them such as *M. stenopetala* and *R. officinalis* exhibited significant antibacterial activity indicating their therapeutic potentials against MRSA and are discussed in detail below. In the present study, the antibiosis of plant extracts positively skewed towards highly and moderately polar solvents such as ethanol, ethyl acetate, and methanol as per the activity indices obtained. The variation in the antibacterial activities noticed is multifactorial and could be due to the difference in the number of constituents in the parent plants or the extent of polarity and solubility related to different solvents. Similar trends were reported in earlier studies (Das et al., 2010; Tiwari et al., 2011; Sasidharan et al., 2011; Seleshe and Kang, 2019).

#### 4.1.1 *Anti-staphylococcal activity of M.* stenopetala

Among the five medicinal plants screened, *M. stenopetala* recorded the widest and the highest range of anti-staphylococcal activity. Ethanolic and ethyl acetate extracts of *M. stenopetala* efficiently repressed the growth of all MRSA tested. Retrospective analysis showed that the antibacterial activity of *M. stenopetala* was known as early as the 1980s (Eilert et al., 1981). For instance, Biffa (2005) stated that the methanolic leaf extract of *M. stenopetala* having a concentration of 1.2 g/ml showed significant inhibitory activity against *S. aureus.* Further, it has been reported that the ethanolic leaf extract (1 mg/ml) of *M. stenopetala* exhibited antibiosis against *S. aureus* (Seleshe and Kang, 2019). However, in a previous study, extracts prepared from leaves were ineffective against *S. aureus* (Chekesa and Mekonnen, 2015). The variation in the antimicrobial activities may be attributed to differences in the time of harvest (Celikel and Kavas, 2008), the developmental stage of plants (Ruberti and Baratta, 2000) and the method of extraction (Manilal and Idhayadhulla, 2014). Prior studies affirmed the antibacterial activities of *M. stenopetala* against *S. aureus* (Tesemma et al., 2013; Raghavendra et al., 2016; El-Fadl et al., 2017), however, the antibiosis against biofilm-forming MRSA has seldom been reported from Ethiopia. In comparison to our study, 80 % methanolic extract of *M. stenopetala* seeds showed a much lower MIC value of 125 μg/ml against *S. aureus* (Chekesa and Mekonnen, 2015). Recently, Seleshe and Kang (2019) reported the inhibitory effect of chloroform extract of *M. stenopetala* on the growth of *S. aureus* at 125 μg/ml. On the other hand, MIC and MBC values obtained from the present study were comparatively higher than the previously reported ones. For instance, El-Fadl et al. (2017) observed a MIC value of 31.25 (μg/ml) in the case of methanolic leaf extract of *M. stenopetala* against *S. aureus.* MIC and MBC values would have been much less if the purified constituents of plant extract were used. Literature shows that *M. stenopetala* is recognized as one of the most promising plants for the production of bioactive metabolites with a wide array of proposed applications (Mekonnen, 2002).

#### 4.1.2 Anti-staphylococcal activity of *R. officinalis*

*Rosmarinus officinalis* also recorded very high activity against the MRSA. The highest activity was observed in the case of ethanolic extract followed by ethyl acetate and methanolic extracts. This result hints at the traditional use of *R. officinalis,* since it produced a higher inhibitory zone. The antibacterial activity of *R. officinalis* against MRSA has been congruent with the findings of prior studies (Jarrar et al., 2010; Alzoubi et al., 2014; Endo et al., 2018). A previous study reported that the hydroalcoholic extract of *R. officinalis* produced MIC values ranging between 15.6 and 62.5 μg/ml against MRSA (Endo et al., 2018). Besides, MICs and MBCs of ethanolic extracts of *R. officinalis* sourced from Saudi Arabia fell in the range between 0.125 and 0.5 mg/ml (Alzoubi et al., 2014). In contrast, the findings of the present study revealed wide variations in the MIC and MBC values. The higher MIC and MBC values observed in this study could be due to the crude nature of extracts since it comprised a larger number of assorted secondary metabolites with or without antimicrobial potentials. In comparison to our study, a study done in Palestine reported higher MIC values corresponding to ethanolic extract of *R. officinalis* (in the range of 0.39–3.13 mg/ml against MRSA) (Jarrar et al., 2010).

The exact mechanism of action of antibacterial constituents in the crude extract of *M. stenopetala* and *R. officinalis* against MRSA was not done in the present study. Nevertheless, based on the results, it could be extrapolated that the mechanism of antibacterial activities of *M. stenopetala* and *R. officinalis* are bacteriostatic rather than bactericidal because of the ratio of MBC/MIC which was ≥2 (Manilal et al., 2010). It has been envisaged that bioactive components could interrupt the permeability of the cytoplasmic membrane of bacteria, thereby inhibiting its growth (Zhao et al., 2000). The overall findings supported the existence of anti-bacterial metabolites in the crude extract of *M. stenopetala* and *R. officinalis*. Clinically, potent antibiotics with minimum side effects to manage MRSA associated infections have not been developed so far. The findings of the present study can be correlated to the possible use of *M. stenopetala* and *R. officinalis* as better sources of anti-bacterial to combat MRSA and the replacement of conventional antibiotics.

### 4.2 Anti-biofilm potential of *M. stenopetala* extract

Biofilms provide a protective shield for the MRSA to resist antibiotic therapy. A notable finding of our study is that the ethanolic extract of *M. stenopetala*, efficiently inhibited the biofilm-formation of poly-culture of MRSA. It could be inferred that the inhibitory activity exhibited by *M. stenopetala* is due to its ability to synthesize metabolites which can prevent biofilm formation. However, this hypothesis needs to be examined further by advanced qualitative and quantitative analyses. A literature survey indicates that the antibiofilm activity of *M. stenopetala* against MRSA is scanty. However, the antibiofilm potential of flavonoids extracted from *M. oleifera* seed coat against *S. aureus* is reported from India (Onsare and Arora, 2015). Our results pertaining to antibiofilm activity agree with previous works done in other species of terrestrial plants from different locales of the world. For instance, a study from India reported that the root extract of *Vetiveria zizanioides* showed a concentration-dependent reduction in biofilm formation of MRSA (Kannappan et al., 2017). Similarly, in another study done in Brazil, it was found that dichloromethane extract of *Piper regnellii* deflates biofilm formation (Lara et al., 2017). Likewise, polyphenolic extracts of *Opuntia ficus-indica* from Italy remarkably inhibited biofilm formation by MRSA (Blando et al., 2019). *M. stenopetala* is one of the most widely and commonly consumed medicinal plants in Ethiopia.

### 4.3 GC-MS analysis of *M. stenopetala* extract

The antibacterial activity of *M. stenopetala* was found to be higher than that of all other plants studied. Therefore, the chemical composition of *M. stenopetala* was studied further in detail by GC-MS analysis. The antimicrobial activities of individual constituents are not elucidated. However, GC-MS analysis of the ethanolic extract showed the presence of 24 peaks. Among them, some of the compounds such as oleic acid, tetradecanoic acid, octadecanoic acid, n-hexadecanoic acid, phenol, 2,4-bis(1,1-dimethyl ethyl), beta.-l- rhamnopyranoside, 5-o-acetyl-thio-octyl, di-(2-ethylhexyl)phthalate and phytol were the prominent antimicrobial compounds. These compounds are already reported in other plants and were found to be active against microbes (Eklund and Gabig, 1990; Dilika et al., 2000; Agoramoorthy et al., 2007; Habib and Karim, 2009; Pu et al., 2010; Tatismo et al., 2012; Mujeeb et al., 2014; Pasmavathi et al., 2014; Swamy and Sinniah, 2015; Patra et al., 2015; Canli et al., 2016; Aabubakar and Majinda, 2016; Lofy et al., 2018; Gadhi et al., 2018). The most interesting finding in this study was the detection of the anti-quorum sensing and anti-biofilm forming compound, phenol, 2,4-bis(1,1-dimethyl ethyl) (Padmavathi et al., 2014). It has been reported that phenol, 2,4-bis(1,1-dimethyl ethyl) isolated from marine bacteria inhibits the formation of quorum sensing mediated biofilm in the uropathogen, *Serratia marcescens*. In addition, a compound, 2-(2′,4′,4′,6′,6′,8′,8′-heptamethyltetrasiloxan-2′-yloxy)-2,4,4 identified in the present study has been previously found in the hexane extract of *Halimeda* sp. which exhibited antibiofilm potential (Canli et al., 2016). However, siloxanes are known to be purely synthetic in its origin. The present study also showed the presence of an anti-inflammatory compound, propanoic acid, 3,3′-thiobis-, diethyl ester in the extract of *M. stenopetala* which could be useful in addressing the inflammatory response to infections (Eklund et al., 1990). The results of the GC-MS analysis envisaged that the antibacterial activity displayed by the *M. stenopetala* extract could be due to the presence of certain specific antimicrobial constituents, or could be attributed to the synergistic activity of all constituents. However, this hypothesis needs to be delved further by bioassay-guided fractionation followed by Fourier-transform infrared spectroscopy and nuclear magnetic resonance analysis.

In Ethiopia, the prevalence of MRSA is slowly rising in the past few decades. In most of the hospitals, antibiotic treatment is not streamlined as per the microbiological culture data. Consequently, MRSA strains are becoming resistant not only to β-lactams but also to multiple antimicrobial agents, such as macrolides, aminoglycosides, and fluoroquinolones (Eshetie et al., 2016; Mama et al., 2018; Manilal et al., 2019). In the present study, two antibiotics such as vancomycin and clindamycin were used as positive controls. Vancomycin is an effective drug for the treatment of MRSA. However, its toxic side effects, need for an intravenous administration and regular monitoring of its level in blood are the major setbacks. Recently, strains of MRSA are showing resistance or reduced susceptibility to vancomycin (Eshetie et al., 2016). Besides, a rising trend in the resistance of MRSA to clindamycin has also been reported from the country (Eshetie et al., 2016). Therefore, novel classes of antibiotics are indispensable to combat the newly emerging multidrug-resistant bacteria like MRSA.

## 5 Conclusion

The prevalence of anti-staphylococcal activity observed in the extracts of *M. stenopetala* provides credible evidence that this plant is a potential source of anti-bacterial metabolites. Results of anti-biofilm assay showed that the extract of *M. stenopetala* efficiently inhibited the growth of MRSA in the preformed biofilm matrix. Hence, it could be inferred that the bioassay-guided fractionation and purification of *M. stenopetala* would bring out new classes of anti-biotic leads. Besides, the actual mechanism of such a broad spectrum of anti-staphylococcal activity exhibited by *M. stenopetala* must be studied in detail.

## Declarations

### Author contribution statement

Aseer Manilal: Conceived and designed the experiments; Performed the experiments; Analyzed and interpreted the data; Wrote the paper.

Kuzhunellil Raghavanpillai Sabu: Analyzed and interpreted the data; Wrote the paper.

Misgun Shewangizaw, Addis Aklilu, Mohammed Seid, Behailu Merdikios, Behailu Tsegaye: Performed the experiments; Analyzed and interpreted the data.

### Funding statement

This work was supported by Research Directorate, College of Medicine and Health Sciences, Arba Minch University (GOV/AMU/TH.3.2/CMHS/MLS/01/09).

### Competing interest statement

The authors declare no conflict of interest.

### Additional information

No additional information is available for this paper.
